# Intranasal Fentanyl for Procedural Analgesia in Preterm Infants

**DOI:** 10.3389/fpain.2021.815014

**Published:** 2022-01-24

**Authors:** Charles Cheng, Najla Tabbara, Carol Cheng, Vibhuti Shah

**Affiliations:** ^1^Department of Pediatrics, Mount Sinai Hospital, Toronto, ON, Canada; ^2^Department of Pharmacy, Mount Sinai Hospital, Toronto, ON, Canada; ^3^Department of Nursing, Mount Sinai Hospital, Toronto, ON, Canada

**Keywords:** fentanyl, administration, intranasal, pain, procedural, preterm infants

## Abstract

**Background:**

Despite the availability of evidence-based analgesic strategies, neonatal pain management continues to be suboptimal. Intranasal (IN) fentanyl is an alternative pharmacotherapy for procedural pain in neonatal units. The objective was to evaluate the effectiveness and safety of IN fentanyl for procedural pain in preterm infants.

**Methods:**

A retrospective cohort study was conducted in infants who received IN fentanyl between May 2019 and December 2020 at an academic neonatal intensive care unit. Main outcome measures were pain responses, physiological parameters before and up to 60 min after IN fentanyl administration, and adverse events. Paired *t*-test and analysis of variance were used to compare pain scores and physiological parameters, respectively.

**Results:**

Thirteen infants received IN fentanyl on 22 occasions. Median (interquartile range [IQR]) gestational age and birthweight were 27 (25, 27.6) weeks and 850 (530, 1,030) grams, while median (IQR) post-menstrual age and weight were 30.9 (28.9, 32.9) weeks and 1,280 (945, 1,623) grams at the time of IN fentanyl administration. IN fentanyl was most used for lumbar puncture (55%) followed by insertion of epicutaneo-caval catheters (27%). There was a difference between the mean pre- and post-procedure Premature Infant Pain Profile scores of 1.3 (95% CI = 0.07, 2.5; *p* = 0.04). Physiological parameters did not differ before and up to 60 min post IN fentanyl administration (*p* > 0.05). Two adverse events (one apnea and one desaturation) were noted.

**Conclusion:**

In our limited experience, IN fentanyl appears to be an alternative pharmacotherapy for procedural pain management in the absence of intravenous access in preterm infants.

## Introduction

Infants admitted to the neonatal intensive care unit (NICU) experience as many as 7 to 17 painful procedures per day, with preterm infants (< 37 weeks) at the highest risk of being exposed to greater numbers of painful procedures (1). Repeated or cumulative exposure to pain is associated with alteration in pain sensitivity and behavioral responses, sub-optimal brain development, and adverse neurodevelopmental outcomes (2, 3). Despite these adverse consequences of pain and the availability of evidence-based non-pharmacological and pharmacological strategies to prevent and minimize pain, their use in the NICUs remains variable and suboptimal (4–7). Potential reasons for inadequate procedural analgesia include the perception that the pain is short-lived and concerns regarding the adverse effects of analgesics, especially opioids (8, 9). For infants with intravenous (IV) access, pharmacological strategies for management of moderate to severe procedural pain include the use of opioids such as morphine and fentanyl (5–7). In the absence of IV access, the intranasal (IN) route is an alternative mode to deliver opioids. It offers rapid and reliable systemic drug absorption with minimal discomfort to patients (10, 11). However, the literature around IN fentanyl use in the NICU setting remains scarce.

Four retrospective studies on the use of IN fentanyl in newborns and infants have been published thus far (12–15) (Table 1). Except for the study by Harlos et al. that prescribed IN fentanyl in the palliative care setting (12), the remaining studies evaluated its use for procedural pain in the NICU (13–15). The sample size in these studies ranged from 2 to 54 patients with mean/median gestational age (GA) at birth ranging from 26 to 35 weeks (13–15). Infants < 28 weeks GA are considered extremely preterm while those between 28 and 32 weeks GA are considered very preterm. The most common procedures for IN fentanyl use were intubation and insertion of epicutaneo-caval catheters (13–15). The dose of IN fentanyl ranged from 0.5 to 2 μg/kg (13–15) and was administered using a mucosal atomization device in the study by McNair et al. (13) or directly instilled into the infants' nares in the studies by Kaushal et al. and Ku et al. (14, 15). Sucrose was administered to all patients in conjunction with IN fentanyl in the study by McNair et al. (13) and IN midazolam was administered concurrently with IN fentanyl in almost half of the 67 events in the study by Kaushal et al. (14). No major adverse events were attributed to IN fentanyl use in the published studies (12–15).

**Table 1:** Summary of the literature.

**Study**	**Population, sample size and indication**	**Dose of IN fentanyl**	**Outcomes**
Harlos et al. (12)	GA range = 24, 41.7 weeks; *N* = 11; Palliative care	Dose = 1–2 μg/kg × 3 doses within 30 min followed by reassessment	No adverse events reported
McNair et al. (13)	Mean (SD) GA = 31.8 (4.1) weeks; Mean (SD) PMA = 35.6 (4.6) weeks; *N* = 23; Procedural pain	Dose = 1.5 μg/kg; 5 infants required a second dose	PIPP scores Mean [SD]: Pre-procedure = 4.8 (3.2) During procedure = 4.3 (1.8) Post-procedure = 3.6 (1.5); Cardio-respiratory depression in 6 patients (not attributed to IN fentanyl)
Kaushal et al. (14)	Median (IQR) GA = 26 (24.1, 36.1) weeks; Median (IQR) PMA = 38.1 (33.1, 45.4) weeks; *N* = 54; Procedural pain and sedation	Dose = Mean of 1.46 μg/kg (range 0.5–2 μg/kg); 6 procedures required a second dose; IN midazolam was co-administered in 32 cases	No adverse events attributed to IN fentanyl
Ku et al. (15)	Mean GA = 35 weeks; *N* = 2; Procedural pain	Dose = 1–2 μg/kg; No second dose administered	No adverse events attributed to IN fentanyl

*GA, gestational age; IQR, interquartile range; IN, intranasal; N, number of patients; PIPP, Premature Infant Pain Profile; PMA, post-menstrual age; SD, standard deviation*.

While the administration of fentanyl via the IN route is currently not endorsed by neonatal and pediatric drug information resources (for infants and children < 10 kg) or Canadian drug monographs (16–18), it has been used in the neonatal population for procedural pain and as part of palliative care (12–15). It offers several advantages including quick onset (within 5–10 min) and short duration of action, allowing for early targeted analgesia with fewer adverse effects and has been used widely in children in emergency settings (19, 20). In May 2019, a practice guideline on the use of IN fentanyl was added to the repertoire of pain management strategies in the NICU at our institution. The objective of our study was to describe our experience with IN fentanyl, specifically evaluating its effectiveness and safety profile.

## Methods

The study protocol was approved by the institutional Research Ethics Board. A retrospective chart review of infants who received IN fentanyl at Mount Sinai Hospital between May 2019 and December 2020 was undertaken. IN fentanyl could be used for procedural pain management and for palliative care when IV access was not established or analgesia was needed for a short period of time. Contraindications to its use include the presence of choanal atresia, nasal mucosal erosion, or epistaxis. Prior to implementation of the guideline, education sessions were held for healthcare professionals (nurses and medical personnel) regarding the indications for IN fentanyl and method of administration. Infants who received IN fentanyl were identified from the pharmacy database and included in the study. IN fentanyl was administered at a dose of 1.5 μg/kg using a mucosal atomization device which converts the fentanyl solution into 30 μm particles. Using a quick push on the syringe plunger, the dose of fentanyl is dispersed in mist form into the nasal cavity, further enhancing its absorption into the vascular bed of the nasal mucosa. The size of the mucosal atomization device does not limit its usage in extremely preterm infants as it needs to be placed at the nares to allow the mist to be dispensed. After 5 min, a second dose could be administered based on the bedside nurse's clinical assessment (maximum two doses per procedure).

Data on infants who received IN fentanyl were collected from the hospital medical records using a pre-designed data collection form retrospectively. Data included demographics [GA at birth, birthweight (BW), sex, post-menstrual age (PMA), and weight] and type of respiratory support at the time of IN fentanyl administration. In addition, data on the type of procedure, number of attempts (> one) and number of IN fentanyl doses per procedure were collected. Effectiveness of IN fentanyl was assessed using the Premature Infant Pain Profile (PIPP) score by the bedside nurse. The pre-procedure PIPP score was recorded just before the administration of the first dose of IN fentanyl and commencement of the painful procedure while the post-procedure PIPP score was recorded within 30 s of successful completion of the procedure. The PIPP score ranges from 0 to 21. A PIPP score of 0–6 suggests minimal or no pain, 7–12 indicates moderate pain and a score ≥ 13 is interpreted as severe pain (21). The physiological parameters of heart rate, respiratory rate, oxygen saturation, and fraction of inspired oxygen (FiO_2_) at baseline (just before IN fentanyl administration) and at pre-specified intervals (15, 30, 45, and 60 min) after IN fentanyl administration were also recorded. Adverse events were defined as apnea (cessation of breathing for >20 s), bradycardia (heart rate < 100 beats/minute), desaturation (oxygen saturation < 80%), and chest wall rigidity and were monitored for 60 min after IN fentanyl administration.

Statistical analyses were performed using R statistical software version 4.0.4 (R Foundation for Statistical Computing, Vienna, Austria). Descriptive statistics were used to summarize patient demographic and clinical characteristics. Continuous data were reported as mean and standard deviation (SD) or median and interquartile range (IQR) as appropriate while categorical data were reported as number and percentage. Changes in pain scores and physiological parameters before and after IN fentanyl administration were compared using paired *t*-test and analysis of variance, respectively. All reported *p*-values are two-sided and *p* < 0.05 were considered statistically significant.

## Results

Thirteen infants received IN fentanyl on 22 occasions (Table 2). The median (IQR) GA at birth and BW was 27 (25, 27.6) weeks and 850 (530, 1,030) grams, respectively, and eight (62%) infants were male. For 17 events (77%), infants were receiving various forms of non-invasive ventilatory support including continuous positive airway pressure, non-invasive positive pressure ventilation, and non-invasive neutrally adjusted ventilatory assist. IN fentanyl was most used for lumbar puncture (55%) followed by insertion of epicutaneo-caval catheters (27%) and endotracheal intubation (18%). A second dose of IN fentanyl was administered in three infants (four events [18%]) at the discretion of the bedside nurse for agitation.

**Table 2:** Clinical characteristics of patients at IN fentanyl administration.

**Variable**	**Total events** **(*N* = 22)**
Post-menstrual age (weeks)	30.9 (28.9, 32.9)
Weight at the time of IN fentanyl administration (grams)	1,280 (945, 1,623)
Type of respiratory support Invasive Non-invasive	5 (23%) 17 (77%)
Type of procedure Lumbar puncture Epicutaneo-caval catheter insertion Endotracheal intubation	12 (55%) 6 (27%) 4 (18%)
Second dose of IN fentanyl administered	4 (18%)

*Results are presented as median (interquartile range) or number (percentage)*.

*IN, intranasal*.

**Figure 1:**
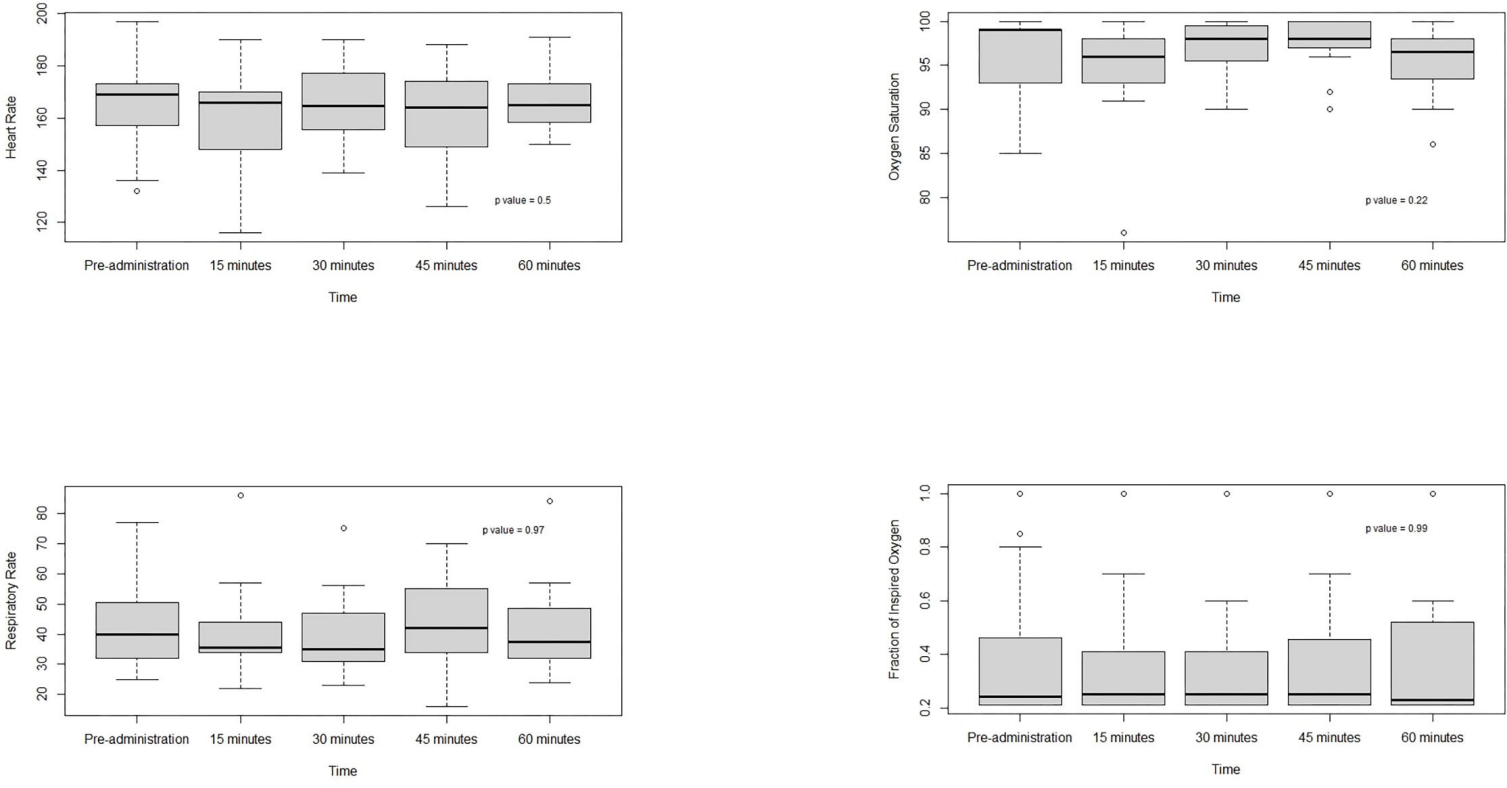
Physiological parameters before and up to 60 min after IN fentanyl administration.

The mean (SD) pre-and post-procedure PIPP scores were 5.4 (2.4) and 4.2 (1.6), respectively. There was a statistically significant difference in the mean PIPP score of 1.3 (95% CI 0.07, 2.5; *p* = 0.04). Physiological parameters before and up to 60 min post-administration of IN fentanyl were not significantly different (*p* > 0.05) (Figure 1). Of the 22 events reported, in 10 events the procedure had to be repeated more than once (lumbar puncture [*n* = 7], epicutaneo-caval catheter insertion [*n* = 2], and endotracheal intubation [*n* = 1]). The post-procedure PIPP score was always recorded after successful completion of the procedure.

Two adverse events were recorded. One infant weighing 710 g with PMA of 27.7 weeks had respiratory deterioration with increasing FiO_2_ and hence a decision was made to intubate. Two minutes after IN fentanyl administration, the infant had an apneic episode requiring positive pressure ventilation. The second infant weighing 880 g with PMA of 26.9 weeks needed a lumbar puncture for suspected sepsis. This infant had an episode of desaturation 45 min after IN fentanyl administration and required increase in oxygen concentration briefly. There were no reports of bradycardia or chest wall rigidity.

## Discussion

In this small study of preterm infants, we were able to demonstrate the effectiveness of IN fentanyl in reducing procedural pain as assessed by PIPP scores. Our practice consisted of administration of IN fentanyl as the only analgesic without the use of adjuncts such as sucrose or IN midazolam. Our results are consistent with the PIPP scores from the study by McNair et al. in which the mean (SD) pre-procedure score was 4.8 (3.2) and the post-procedure score was 3.6 (1.5) (13). Despite exhibiting physiological and behavioral responses to stress and painful stimuli in the NICU environment, preterm infants have diminished mechanisms to modulate pain compared to older infants (22–24). Other potential reasons for lower PIPP scores may include the inability of critically ill preterm infants to mount responses, and inaccurate pain assessments due to health care professionals' potential lack of knowledge and understanding of neonatal pain or inappropriate use of the PIPP tool (9, 25–28). There were no differences noted in physiological parameters up to 60 min after its administration and there were no major adverse events.

In contrast to infants in the NICU, IN fentanyl has been well-adopted in the emergency department when children present with moderate to severe pain secondary to fractures or burns. In the non-neonatal pediatric population, its effectiveness is well-established with an improved safety profile compared to IV opioid administration (20, 29). As an alternative route of drug administration, IN fentanyl is a highly lipid soluble medication with rapid absorption by the highly vascular nasal mucosa with the potential of being transported directly into the cerebrospinal fluid (30, 31). It has an approximate bioavailability of 70% and avoids first pass metabolism by the liver (32). It provides timely analgesia without delaying procedures and decreases the requirement of invasive IV line placements which may be difficult to secure. Considering its advantages and ease of administration, IN fentanyl is an appealing pharmacological alternative in the management of neonatal procedural pain. The results of our study add to the growing body of literature supporting IN fentanyl use in the preterm population.

Limitations of our study include a small sample size over a 20-month period, absence of a control group, and potential risk for selection bias as not all eligible patients may have received the therapy. We aimed to reduce this bias by providing education to healthcare professionals regarding infants who would benefit from this intervention but our sample may not be fully representative of our NICU patients even though our findings are consistent with other studies of IN fentanyl in the neonatal population. Another limitation is the timing of the post-procedure PIPP score in infants who received two doses of IN fentanyl. The time interval between the second dose and post-procedure PIPP score was not captured. We did not assess the perception (beliefs and attitudes) of healthcare professionals regarding the use of IN fentanyl for procedural pain and this should be evaluated in future studies as this information may be relevant for its adoption in clinical practice. Engagement and satisfaction from parents and caregivers should also be considered when assessing IN fentanyl use in patients in the NICU.

Randomized controlled trials are considered the gold standard to assess the effectiveness and safety of an intervention and future research on IN fentanyl should be undertaken using this study design with adequate sample size. Pharmacokinetic and pharmacodynamic studies on IN fentanyl in preterm infants will also be beneficial to optimize drug dosing and delivery and minimize adverse effects. Qualitative studies assessing healthcare providers' perceptions, facilitators, and barriers to IN drug administration should be examined as physicians and nurses play a key role in pain management. While awaiting further research, IN fentanyl provides an alternative therapeutic option to manage procedural pain in neonates when IV access is not available.

## Conclusion

IN fentanyl appears to be an alternative pharmacotherapy for procedural pain management in the absence of IV access in preterm infants.

## Data Availability Statement

The data analyzed in this study is subject to the following licenses/restrictions. The dataset for this study is not available without institutional Research Ethics Board approval. Data were collected by chart review using medical records from Mount Sinai Hospital, Toronto, Ontario, Canada. Requests to access these datasets should be directed to Vibhuti Shah, Vibhuti.Shah@sinaihealth.ca.

## Ethics Statement

The studies involving human participants were reviewed and approved by Mount Sinai Hospital Research Ethics Board. Written informed consent from the participants' legal guardian/next of kin was not required to participate in this study in accordance with the national legislation and the institutional requirements.

## Author Contributions

NT, CaC, and VS: study design. ChC and CaC: data collection. NT and VS: statistical calculations and analyses. ChC and NT: drafting of the manuscript. ChC, NT, CaC, and VS: critical review and editing of the manuscript. All authors contributed to the article and approved the final manuscript.

## Conflict of Interest

The authors declare that the research was conducted in the absence of any commercial or financial relationships that could be construed as a potential conflict of interest.

## Publisher's Note

All claims expressed in this article are solely those of the authors and do not necessarily represent those of their affiliated organizations, or those of the publisher, the editors and the reviewers. Any product that may be evaluated in this article, or claim that may be made by its manufacturer, is not guaranteed or endorsed by the publisher.
